# High plant diversity alleviates the negative effects of nitrogen deposition on soil nitrogen cycling multifunctionality

**DOI:** 10.3389/fmicb.2025.1596515

**Published:** 2025-05-14

**Authors:** Zhuo Li, Xiaowei Liu, Minghui Zhang, Bin Zhang, Tao Zhang, Cameron Wagg, Fu Xing

**Affiliations:** ^1^Liaoning Key Laboratory of Development and Utilization for Natural Products Active Molecules, School of Chemistry and Life Science, Anshan Normal University, Anshan, Liaoning, China; ^2^Key Laboratory of Vegetation Ecology, Jilin Songnen Grassland Ecosystem National Observation and Research Station, Institute of Grassland Science, Northeast Normal University, Ministry of Education, Changchun, China; ^3^Remote Sensing Laboratories, Department of Geography, University of Zürich, Winterthurerstrasse, Zürich, Switzerland; ^4^Fredericton Research and Development Centre, Agriculture and Agri-Food Canada, Fredericton, NB, Canada

**Keywords:** nitrogen deposition, plant diversity, soil microbial carbon limitation, soil nitrogen cycling multifunctionality, global change

## Abstract

**Introduction:**

Changes in plant diversity and increased atmospheric nitrogen deposition independently influence soil nitrogen cycling in terrestrial ecosystems. However, the interactive effects of plant diversity and nitrogen deposition on soil nitrogen cycling multifunctionality (NCMF) in grassland ecosystems remain poorly understood.

**Methods:**

We conducted a fully factorial microcosm experiment to quantify the responses and underlying mechanism of soil NCMF to nitrogen addition (0, 5, and 10 g N m^−2^ yr.^−1^) and plant diversity gradients (1, 3, and 6 species).

**Results:**

Our results revealed a significant interactive effect between plant diversity and nitrogen addition on soil NCMF. Specifically, high plant diversity alleviated the negative effects of nitrogen addition on soil NCMF. The addition of nitrogen reduced the soil pH, which imposed microbial stress by limiting carbon availability. In contrast, higher plant diversity increased soil organic matter via below-ground carbon inputs, thereby reducing the soil carbon limitation of microorganims and enhancing the soil NCMF.

**Discussion:**

Overall, our findings suggest that maintaining or enhancing plant diversity in grasslands could be a key strategy to mitigate the adverse effects of atmospheric nitrogen deposition on soil nitrogen cycling, highlighting the crucial role of plant diversity in regulating ecosystem nutrient cycling under global change.

## 1 Introduction

Nitrogen is a critical nutrient for both plants and soil microorganisms (Wang et al., 2023). Nitrogen cycling, as an essential component of biogeochemical cycles, plays a pivotal role in supporting organic growth and maintaining ecosystem stability (Chen and Chen, 2021), and has been demonstrated to be strongly linked to changes in plant diversity and nitrogen deposition (Hallin et al., 2009; Maestre et al., 2011). However, previous studies have primarily examined the individual effects of plant diversity and nitrogen deposition on nitrogen cycling (Lu et al., 2010; Byrnes et al., 2014), and their interactive effects remain unclear, hampering our ability to understand the responses and adaptations of ecosystem nutrient cycling to plant diversity and nitrogen deposition.

Many studies have shown that plant diversity and nitrogen deposition significantly affect soil nitrogen cycling functions, but the results are inconsistent. For example, plant diversity is positively correlated with soil ammonium nitrogen availability and nitrogen mineralization rates, whereas soil nitrate nitrogen availability tends to decrease with increasing plant diversity (Oelmann et al., 2011). Moreover, plant species mixtures can increase the abundance of soil nitrifiers and denitrifiers (Shah et al., 2013). Additionally, previous studies have shown that nitrogen addition enhances nitrification by increasing the abundance of ammonia-oxidizing bacteria (AOB) and the *Nitrospira* genus (Ma et al., 2016; Yang et al., 2020). However, other studies have reported the neutral impacts of nitrogen fertilization on nitrogen mineralization rates and the inhibitory effects on denitrification (e.g., *narG* gene abundance) (Tang et al., 2016). Furthermore, leucine aminopeptidase (LAP) and *β*-1,4-N-acetylglucosaminidase (NAG), the hydrolytic enzymes related to nitrogen cycling, have been found to decrease under high nitrogen addition (Cui et al., 2020). These results indicate that the response of individual nitrogen cycling functions to plant diversity and nitrogen deposition significantly differs and that there is a certain trade-off, because soil nitrogen cycling functionality is inherently linked to multiple ecosystem processes rather than isolated functions (Cheng et al., 2022; Wang et al., 2023; Yang et al., 2023). Therefore, assessing single functions cannot fully capture how soil nitrogen cycling responds to environmental changes in real-world scenarios (Cui et al., 2020). This highlights the importance of exploring the relationship between soil nitrogen cycling multifunctionality (NCMF) and environmental conditions in grassland ecosystems.

Increasing attention has been given to the response of soil NCMF to changes in plant diversity and nitrogen deposition. Both plant diversity and nitrogen addition have been shown to independently alter soil NCMF (Cui et al., 2020; Zhang et al., 2021; Freitag et al., 2023). For example, greater plant diversity has been associated with greater soil NCMF, likely due to increased resource heterogeneity, niche differentiation, and complementary resource use among plant species, increasing ecosystem stability and productivity (Byrnes et al., 2014). Similarly, diverse plant communities can also support a wider range of microbial taxa involved in nitrogen cycling, such as nitrifiers, denitrifiers, and nitrogen-fixing bacteria (Chen et al., 2020), thereby enhancing soil NCMF. Nitrogen addition has been shown to increase soil NCMF (Cui et al., 2020) because it provides a readily available nutrient source for soil microbes, stimulating their activity and growth (Li et al., 2020b). However, excessive nitrogen addition can lead to soil acidification, changes in microbial community composition, and potential declines in microbial diversity (Ma et al., 2016), which may negatively affect soil NCMF over time. Nevertheless, the interactive effects of plant diversity and nitrogen addition on soil NCMF remain unclear.

The interactive effects of plant diversity and nitrogen deposition on soil NCMF have not been fully explored. Although separate studies on plant diversity and nitrogen deposition suggest that both factors may influence NCMF through distinct mechanisms (Fornara and Tilman, 2008; Cui et al., 2020; Delgado-Baquerizo et al., 2020; Li et al., 2020b; Freitag et al., 2023). The interactive effects of plant diversity and nitrogen addition on soil NCMF are complex and may depend on several factors, including the magnitude of nitrogen input, the composition of the plant community, and the initial soil nutrient status. The combination of high plant diversity and nitrogen addition may lead to synergistic effects on soil NCMF. For example, diverse plant communities may be better able to utilize added nitrogen, leading to increased soil microbial activity and increased NCMF (Shah et al., 2013; Meyer et al., 2016). Increased nitrogen deposition alters soil environmental conditions, such as the available nitrogen content and soil pH, which directly affect microbial diversity and activity (Wang et al., 2011; Wu et al., 2024), thereby influencing nitrogen cycling processes. However, the presence of diverse plant species may also buffer against the negative effects of nitrogen addition, such as soil acidification, by maintaining a more stable soil environment (Turnbull et al., 2012; van der Plas, 2019; Xu et al., 2022). Higher plant diversity has been associated with increased soil microbial biomass and activity due to greater plant-derived carbon inputs and expanded microbial niches (García-Palacios et al., 2010; Chen et al., 2020), which can enhance both mineralization rates and immobilization rates of soil nitrogen (Fornara and Tilman, 2008; Meyer et al., 2016). Additionally, increased soil carbon resulting from increased plant diversity can meet the energy demands of carbon-intensive microbial processes and nitrogen cycling functional gene expression, thereby accelerating soil nitrogen turnover (Shu et al., 2011; Cui et al., 2021). Nitrogen addition may reduce the positive effects of plant diversity on soil NCMF. For instance, nitrogen addition leads to the dominance of a few plant species that outcompete others, which could reduce plant diversity and, in turn, negatively impact soil microbial diversity and function (Chen et al., 2020). Furthermore, complementarity in resource use and or facilitative interactions were the main drivers of increased productivity at higher levels of species richness (van Ruijven et al., 2005). Higher plant diversity communities have varied root architectures and growth forms, which could lead to more efficient exploration of soil volume (Xu et al., 2022). In contrast, nitrogen addition would homogenize soil conditions and reduce the spatial heterogeneity of soil resources (He et al., 2021), potentially reducing the positive effects of plant diversity on soil NCMF. Nevertheless, it remains unclear whether and to what extent plant diversity modulates the effects of nitrogen addition on soil NCMF.

In this study, we conducted a two-year microcosm experiment to investigate the effects and potentially microbial mechanisms of plant diversity and nitrogen addition on soil NCMF. We propose three hypotheses: (1) Plant diversity and nitrogen addition interactively affect soil NCMF (Cui et al., 2020; Delgado-Baquerizo et al., 2020; Li et al., 2020a,b; Freitag et al., 2023); (2) higher nitrogen addition increases soil microbial carbon limitation by reducing soil pH and thus affects soil NCMF (Ma et al., 2016; Yang et al., 2020); and (3) higher plant diversity positively influences soil organic matter and reduces microbial carbon limitation, thereby significantly increasing soil NCMF (Fornara and Tilman, 2008; Meyer et al., 2016; Chen et al., 2020).

## 2 Materials and methods

### 2.1 Study region

We conducted a microcosm experiment at the Jilin Songnen Grassland Ecosystem National Observation and Research Station, which is located in the Songnen grassland, China. The region is characterized by a meadow steppe dominated by *Leymus chinensis*, with widespread salinized soils. The climate is a continental monsoon climate, with a mean annual temperature ranging from 4.6 to 6.4°C and an average annual precipitation of 470.9 mm, 70% of which occurs between June and August (Cui et al., 2020).

### 2.2 Preparation of the plant species pool

We surveyed local plant species in the Songnen grassland and selected six native species that frequently occur in the area: *Leymus chinensis*, *Hierochloe glabra*, *Lespedeza daurica*, *Vicia amoena*, *Carex duriuscula*, and *Kalimeris integrifolia*. These species were used to prepare aseptic seedlings. The sterilized seeds were sown in plastic boxes (20 cm internal diameter, 30 cm depth) and placed in a climate-controlled greenhouse at Northeast Normal University (25°C, 70% relative humidity, 16-h daylight at 5000 lux illumination). Seedlings approximately 5 cm in height were selected and transplanted into microcosms (30 cm internal diameter, 40 cm depth). The homogenized meadow soil used in this study was collected from the Songnen grassland and sieved to remove roots and stones. To minimize interactions with the soil seed bank, the top 5 cm of soil was removed (García-Palacios et al., 2010).

### 2.3 Experimental design

The experiment comprised 120 pots arranged in 24 combinations, representing three nitrogen addition levels (N0: 0, N1: 5, and N2: 10 g NH_4_NO_3_–N m^−2^ yr.^−1^) and three levels of plant diversity (S1: 1 species, S3: 3 species, and S6: 6 species), with five replicates per combination. As soil NCMF tended to saturate at N rates ≥ 10 g m^−2^ yr.^−1^ in this grassland, nitrogen addition was applied at above three levels (Cui et al., 2020). We cultivated monocultures of all six plant species, as well as three-species combinations and mixtures of all six species, resulting in eight distinct plant compositions. Theoretically, there are 20 potential combination models for selecting three species from the six available; however, the experimental workload would be prohibitively large. Therefore, we randomly selected one representative combination model—*L. chinensis* + *L. daurica* + *V. amoena*—to exemplify the three-species combinations. While this selection does not encompass all possible tri-species combinations, it effectively represents the ecological responses associated with other plant assemblages across different families, genera, or functional groups.

Each pot was filled with 21.5 kg of homogenized meadow soil. The pH of the homogenized soil was 8.14, and the NH_4_^+^-N, NO_3_^−^-N, and total carbon (TC) concentrations were 2.53 mg kg^−1^, 0.21 mg kg^−1^, and 4.07 g kg^−1^, respectively. The soil was irrigated to adjust the gravimetric water content to match field conditions. Twelve seedlings selected were planted in each pot, ensuring an equal likelihood of neighboring species across microcosms (Fukami et al., 2001). Pots were randomly arranged in an open-ended greenhouse at the field station. The microcosms were buried with their top edges 3 cm above ground level to prevent surface runoff and left for 30 days to allow plant community establishment. Deionized water was applied weekly to ensure plant survival. In late September, the microcosms were mown to simulate local farming practices, and weeds were removed regularly throughout the experiment.

Nitrogen treatments were applied via the addition of chemically pure NH_4_NO_3_ (35% nitrogen content). The annual fertilization amount was divided into monthly applications during the growing season (mid-June to August) over 2 years. Each NH_4_NO_3_ portion was dissolved in deionized water and evenly sprayed into the pots. The control pots received equivalent amounts of deionized water to account for the water addition effect. In the first year, no plants or soils were harvested to allow for transient responses of the soil and plant properties. Sampling occurred at peak plant biomass in the second year.

### 2.4 Plant sample and soil collection and analyses

The plant and soil samples were collected in August of the second year (peak growing season). The above-ground biomass (AGB) and below-ground biomass (BGB) were harvested from each pot. The plant tissues were heated at 105°C for 30 min to halt metabolic activity, then dried at 65°C and weighed (Rillig et al., 2002).

Soil samples were collected from the upper 10 cm at five points in each pot and mixed into a composite sample. The soil was sieved through a 2 mm mesh to remove roots and divided into three parts for analysis. One part was stored at 4°C for measuring the soil water content (SWC), available nitrogen (NO₃^−^–N and NH₄^+^–N), soil net nitrogen mineralization rate (R_m_), and net nitrogen nitrification rate (R_n_). The second part was air-dried to analyze the soil pH, total nitrogen (TN), soil organic matter (SOM), and TC. The third part was stored at −80°C for qPCR and enzyme activity assays (LAP, NAG, alkaline phosphatase: ALP, and *β*-1,4-glucosidase: βG).

The soil pH was measured via a PHS-3E glass electrode (Leichi, Shanghai, China) in a 1:5 soil-to-water suspension. The SWC was determined by drying the samples at 105°C to a constant weight. Available nitrogen was quantified via a continuous flow analyzer (Futura, Alliance-AMS, France). R_m_ and R_n_ were measured during aerobic incubation (Hart et al., 1994). TN and TC were analyzed via an elemental analyzer (Isoprime 100, Isoprime Ltd., Manchester, United Kingdom). Soil organic carbon was analyzed using a total organic carbon analyzer (vario TOC cube, Elementar, Hanau, Germany) through dry combustion. According to Van Bemmelen factor, soil organic matter content (SOM) = soil organic carbon content × 1.724. Microbial biomass carbon was determined with the chloroform fumigation–extraction method (Cui et al., 2020). Enzyme activities were measured via a microplate fluorometric assay (TECAN Infinite F200, Tecan Group, Switzerland) (Cui et al., 2020).

### 2.5 DNA extraction and qPCR analysis

Soil DNA was extracted using the Power Soil DNA Isolation Kit (MoBio Laboratories, San Diego, CA, United States) according to the manufacturer’s instructions. The purified DNA was stored at −80°C after purification with a DNA purification kit and verification via 2% agarose gel electrophoresis. The abundance of 16S rRNA and 18S rRNA genes was determined using quantitative PCR (qPCR). PCR amplifications were conducted in triplicate 20 μL reaction mixtures, each containing 4 μL of 5 × FastPfu Buffer, 2 μL of dNTPs, 0.8 μL of each primer, 0.4 μL of FastPfu Polymerase, 10 ng of template DNA, and ultra-pure water to adjust the volume to 20 μL. The PCR protocol was as follows: initial denaturation at 95°C for 2 min, followed by 27 cycles of 95°C for 30 s, 55°C for 30 s, and 72°C for 40 s, with a final extension at 72°C for 10 min. The PCR products were then excised from 2% agarose gels and purified using the AxyPrep DNA Gel Extraction Kit (Axygen Biosciences, Union City, CA, United States) according to the manufacturer’s guidelines. The quantification of the abundances of nitrogen cycling microbial genes was performed by a StepOne™ Real-Time PCR System (Applied Biosystems, CA, United States) with a 20 μL reaction volume, including 2 μL DNA templates, 0.4 μL forward and reverse primers, and 10 μL Fast qPCR Master Mix (BBI, Canada). Tenfold serial dilutions of the linearized plasmid DNA were used to establish a standard curve for each gene. Copy numbers per ng DNA were transformed into gene copy numbers per gram of soil using the DNA amount retrieved per gram of soil (Ma et al., 2016). Standard curves were generated via tenfold serial dilutions of linearized plasmid DNA, and gene copy numbers were normalized per gram of soil. Meanwhile, the microbial community structure was assessed by the ratio of fungal gene copies and bacterial gene copies.

### 2.6 Soil microbial carbon limitation

Soil microbial carbon limitation was assessed using vector analysis of enzymatic stoichiometry (Chen et al., 2021). The vector length, calculated as $\sqrt{{\left(\ln\beta G/\ln(\mathrm{NAG}+\mathrm{LAP})\right)}^{2}+{\left(\ln\beta G/\text{lnALP}\right)}^{2}}$ , represents the degree of carbon limitation, with a greater vector length indicating a stronger limitation.

### 2.7 Assessing soil NCMF

Six soil variables related to nitrogen storage and cycling, including soil *nifH*, AOB *amoA*, *nirK*, *nirS*, R_m_, and R_n_, were measured in this study (Maestre et al., 2012; Delgado-Baquerizo et al., 2020; Osburn et al., 2021; Xu et al., 2021). These variables represent the processes of nitrogen cycling and available nutrient supply (Wang et al., 2023). The soil NCMF was calculated using an averaging approach, which has been widely used in multifunctionality analyses (Manning et al., 2018; Cui et al., 2020; Xu et al., 2021; Wang et al., 2023). To obtain the average soil NCMF index, we tested all variables for normal distribution using the Shapiro–Wilk test prior to analysis, and logarithm or square root transformation was performed when necessary. Nine soil variables were standardized and normalized using z-score transformations, and the average of these transformed values was calculated as the final result (Maestre et al., 2012; Valencia et al., 2018).

### 2.8 Statistical analyses

Two-way ANOVA was used to assess the individual and combined effects of plant diversity and nitrogen addition on ecosystem attributes (BGB, AGB, soil pH, SWC, TC, SOM, NO_3_^−^-N, NH_4_^+^-N, TN, the gene abundances of *nifH*, AOB *amoA*, *nirK*, *nirS*, NAG, LAP, ALP, βG, R_m_, R_n_, bacterial abundance, fungal abundance, fungi to bacteria ratio, microbial biomass carbon, microbial carbon limitation, and soil NCMF). Microbial gene abundance was log_10_ transformed before two-way ANOVA. *p* < 0.05 was considered to identify statistically significant differences. Tukey’s HSD *post-hoc* tests were used to evaluate the effects of nitrogen addition and plant diversity on soil NCMF. A piecewise structural equation model was used to test the direct and indirect effects of plant diversity and nitrogen addition on soil NCMF. The model assumes that plant diversity, nitrogen addition, and their interaction alter soil microbial carbon limitation levels via changes in soil pH, BGB, bacterial abundance, and SOM content and that they ultimately influence the soil NCMF. We constructed a *prior* model based on the known effects and potential relationships (Supplementary Figure S1). Fisher’s C statistic and the Akaike information criteria (AIC) were used to assess the goodness of-fit of the model (Shipley, 2013). All statistical analyses were carried out using R software (4.1.2, R Development Core Team, 2015).

## 3 Results

### 3.1 Effects of plant diversity and nitrogen addition on plants and soil properties

Two-way ANOVA revealed that both plant diversity and nitrogen addition significantly influenced SWC, TC, NH_4_^+^–N, and TN concentrations, AGB and BGB (Supplementary Table S1). Additionally, the interaction between plant diversity and nitrogen addition significantly affected on the soil pH, SWC, and TC, soil NO_3_^−^–N, NH_4_^+^–N, and TN concentrations (Supplementary Table S1). Compared with the N0 treatment, the N2 treatment significantly reduced the soil pH under the S3 and S6 treatments but significantly increased the SWC (Table 1). Furthermore, compared with the N0 treatment, the N1 and N2 treatments significantly elevated the soil TC and BGB under the S3 and S6 treatments (Table 1). Under N1 condition, S6 treatment significantly reduced soil pH than the S1 treatment (Table 1). Under N0 and N2 condition, S6 treatment significantly increased SWC than the S1 treatment (Table 1). Under N0, N1 and N2 condition, S6 treatment significantly increased SOM, TC, and BGB than the S1 treatment (Table 1). Under the S1, S3, and S6 conditions, the NO_3_^−^–N concentrations in the N2 treatment were significantly greater than those in the N0 treatment (Table 1). Under N0, N1 and N2 condition, S6 treatment significantly increased the soil NO_3_^−^–N concentrations than the S1 treatment (Table 1). Under the N2 condition, S6 treatment significantly increased the soil NH_4_^+^–N concentrations than the S1 treatment (Table 1).

**Table 1:** Effects of plant diversity (S) and nitrogen addition (N) on plant and soil properties in semi-arid grassland mesocosms.

S	N	pH	SWC (%)	SOM	TC	NH_4_^+^-N	NO_3_^−^-N	TN	AGB	BGB
				(g kg^−1^)	(g kg^−1^)	(mg kg^−1^)	(mg kg^−1^)	(mg kg^−1^)	(g pot^−1^)	(g pot^−1^)
S1	N0	8.41 ± 0.18aAB	4.68 ± 0.45abB	6.01 ± 0.28B	4.06 ± 0.12bC	2.05 ± 0.06aA	0.24 ± 0.05bB	89.34 ± 2.06bC	35.95 ± 1.24bB	57.26 ± 0.86bC
	N1	8.45 ± 0.06aA	4.35 ± 0.51bA	6.56 ± 0.15B	4.48 ± 0.02aB	2.22 ± 0.11aA	0.76 ± 0.01bB	47.22 ± 2.56cC	54.49 ± 3.50bB	68.03 ± 3.49aB
	N2	8.13 ± 0.11bA	5.63 ± 0.13aC	6.59 ± 0.73B	4.47 ± 0.08aC	1.02 ± 0.02bB	8.94 ± 0.62aB	154.28 ± 4.11aA	42.36 ± 1.39aAB	71.16 ± 2.68aC
S3	N0	8.09 ± 0.1bB	6.20 ± 0.22bA	6.66 ± 0.15A	4.42 ± 0.07bB	0.81 ± 0.22bB	0.56 ± 0.06bA	112.32 ± 1.87bB	41.78 ± 0.78B	65.34 ± 1.15bB
	N1	8.41 ± 0.09aA	3.72 ± 0.7cA	7.62 ± 0.51B	4.41 ± 0.12bB	2.63 ± 0.26aA	1.34 ± 0.15bB	151.48 ± 5.86aA	50.97 ± 4.70B	82.81 ± 2.22abB
	N2	6.41 ± 0.07cB	10.08 ± 0.13aB	6.59 ± 0.21AB	6.43 ± 0.06aA	0.56 ± 0.08bC	7.30 ± 0.42aC	85.84 ± 0.44cC	43.80 ± 4.83B	72.21 ± 6.58aB
S6	N0	8.58 ± 0.14aA	6.24 ± 0.21bA	6.96 ± 0.07bA	4.68 ± 0.06bA	0.96 ± 0.09cB	0.57 ± 0.13cA	123.00 ± 2.72aA	58.69 ± 4.13A	85.53 ± 2.37cA
	N1	8.05 ± 0.05bB	1.72 ± 0.42cB	8.55 ± 0.42bA	4.74 ± 0.01bA	1.24 ± 0.04bB	7.82 ± 2.11bA	93.40 ± 5.44cB	64.51 ± 3.87A	106.61 ± 0.39bA
	N2	7.09 ± 0.22cA	10.65 ± 0.07aA	7.18 ± 0.038aA	5.55 ± 0.25aB	2.37 ± 0.07aA	16.74 ± 1.13aA	114.60 ± 4.12bB	62.70 ± 4.78A	92.10 ± 1.47aA

Shown are the mean values and standard error. For each column, Different lower- and upper-case letters indicate significant differences (*p* < 0.05) among different nitrogen addition and plant diversity treatment, respectively (Tukey’s HSD) in semi-arid grassland mesocosms.

### 3.2 Effects of plant diversity and nitrogen addition on soil extracellular and microbial carbon limitation

**Figure 1:**
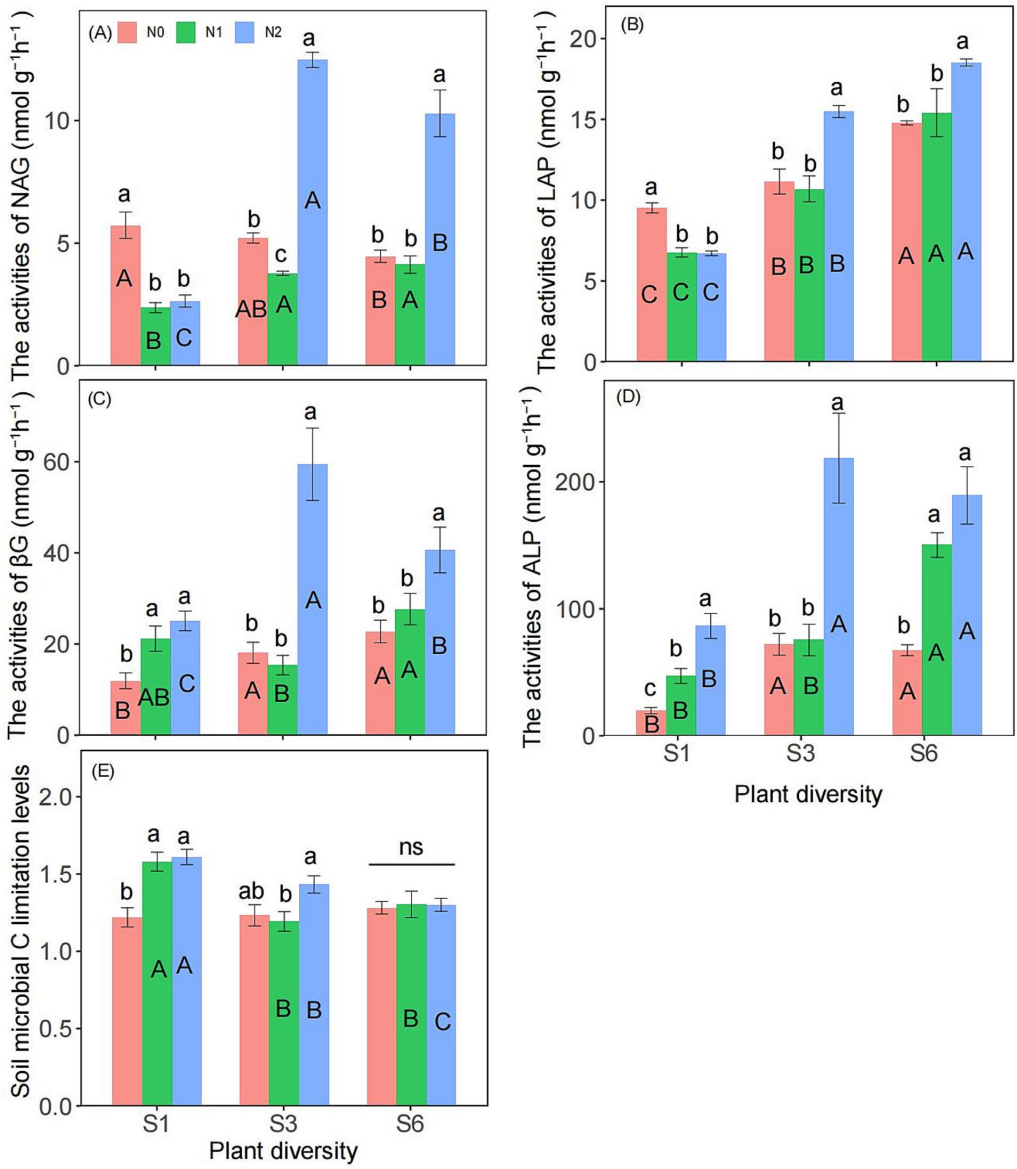
Responses of NAG **(A)**, LAP **(B)**, βG **(C)**, ALP activities **(D)** and soil microbial carbon limitation levels **(E)** to plant diversity and nitrogen addition in semi-arid grassland microcosm. Different lower- and upper-case letters indicate significant differences (*p* < 0.05) among different nitrogen addition and plant diversity treatment, respectively (Tukey’s HSD) in semi-arid grassland mesocosms. ns indicates that the difference is not significant. S1: 1 species, S3: 3 species, S6: 6 species. N0: without nitrogen addition, N1: 5 g N m^−2^ yr.^−1^, N2: 10 g N m^−2^ yr.^−1^.

Two-way ANOVA showed that plant diversity and nitrogen addition significantly influenced the activities of LAP and ALP (Supplementary Table S1). Additionally, the interaction between plant diversity and nitrogen addition significantly affected on the activities of NAG, LAP, βG, ALP, and microbial carbon limitation (Supplementary Tables S1, S2). Under the S1 condition, the N2 treatment significantly reduced the activities of NAG and ALP. However, under the S3 and S6 conditions, N2 significantly increased their activity (Figures 1A,B). Under the S1, S3, and S6 conditions, the activities of βG and ALP in the N2 treatment were significantly greater than those in the N0 treatment (Figures 1C,D). Additionally, under the S1 treatment, the N2 treatment significantly increased soil microbial carbon limitation compared with the N0 treatment, but this effect was not observed under the S6 treatment (Figure 1E). Under N1 and N2 condition, S3 and S6 treatment significantly increased the activities of NAG than the S1 treatment (Figure 1A). Under N0, N1 and N2 condition, S3 and S6 treatment significantly increased the activities of LAP than the S1 treatment (Figure 1B). Under N0 and N2 condition, S6 treatment significantly increased the activities of βG than the S1 treatment (Figure 1C). Under N0, N1 and N2 condition, S6 treatment significantly increased the activities of ALP than the S1 treatment (Figure 1D). Under N1 and N2 condition, S3 and S6 treatment significantly reduced the soil microbial carbon limitation levels than the S1 treatment (Figure 1E).

### 3.3 Effects of plant diversity and nitrogen addition on soil microbial community properties

**Figure 2:**
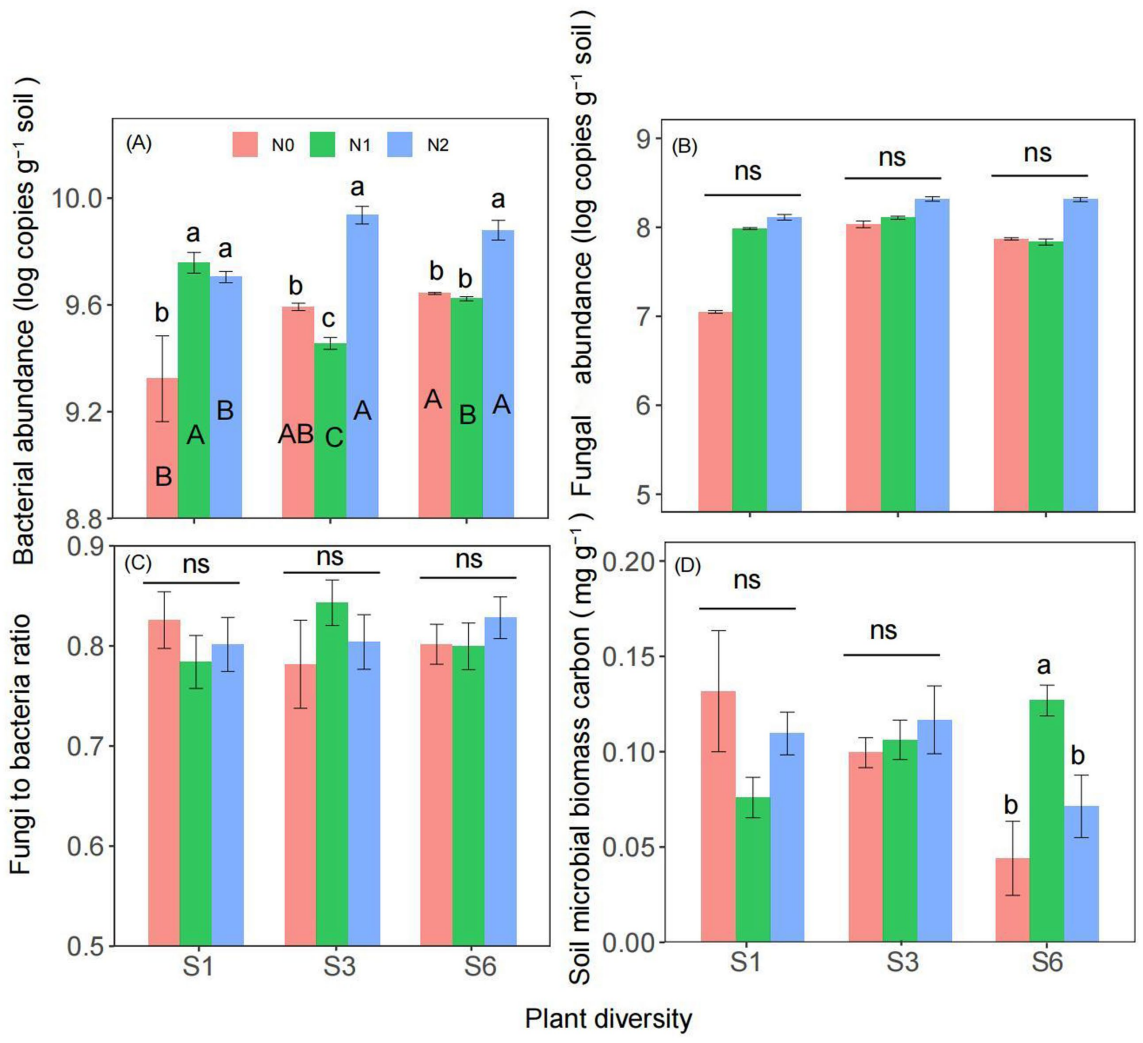
Responses of soil bacterial abundance **(A)**, fungal abundance **(B)**, fungi to bacteria ratio **(C)**, and microbial biomass carbon **(D)** to plant diversity and nitrogen addition in semi-arid grassland microcosm. Different lower- and upper-case letters indicate significant differences (*p* < 0.05) among different nitrogen addition and plant diversity treatment, respectively (Tukey’s HSD) in semi-arid grassland mesocosms. ns indicates that the difference is not significant. S1: 1 species, S3: 3 species, S6: 6 species. N0: without nitrogen addition, N1: 5 g N m^−2^ yr.^−1^, N2: 10 g N m^−2^ yr.^−1^.

Two-way ANOVA revealed that both plant diversity and nitrogen addition significantly influenced soil bacterial abundance (Supplementary Table S1). Moreover, the interaction between plant diversity and nitrogen addition significantly affected on the soil bacterial abundance and microbial biomass carbon (Supplementary Table S1). There was no significant interaction between plant diversity and nitrogen addition on fungal abundance and the fungi to bacteria ratio (Supplementary Table S1). Compared with the N0 treatment, the N2 treatments significantly increased the soil bacterial abundance under the S1, S3 and S6 treatments (Figure 2A). Under N0 and N2 condition, S6 treatment significantly increased the soil bacterial abundance than the S1 treatment (Figure 2A). Compared with the N0 treatment, the N2 treatments have no significantly increased the fungal abundance, fungi to bacteria ratio, and microbial biomass carbon under the S1and S3 treatments (Figures 2B–D).

### 3.4 Effects of plant diversity and nitrogen addition on soil nitrogen cycling functions and NCMF

**Figure 3:**
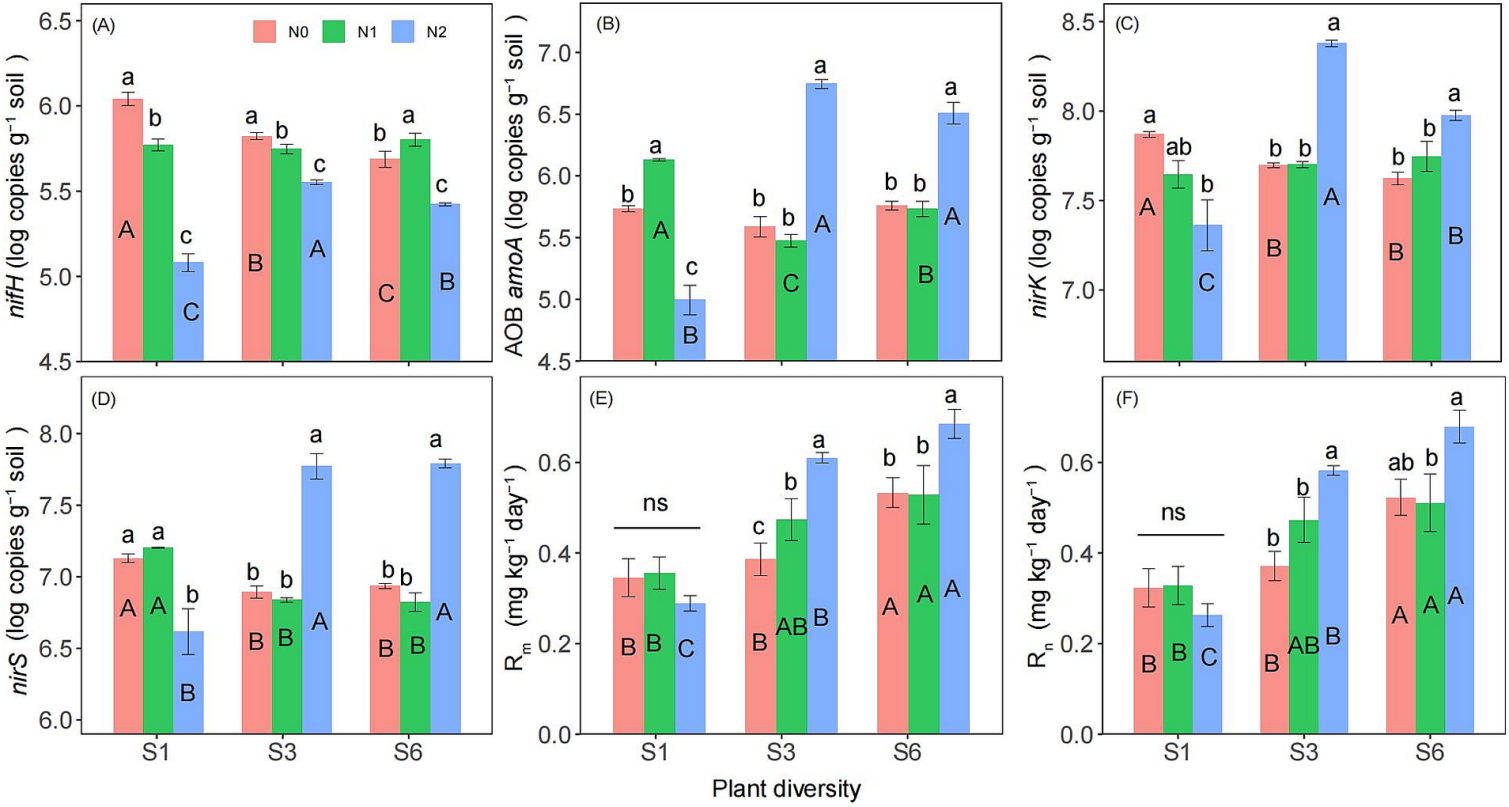
Responses of *nifH* **(A)**, AOB *amoA* **(B)**, *nirK* **(C)**, *nirS* **(D)**, R_m_ **(E)**, and R_n_ **(F)** to plant diversity and nitrogen addition in semi-arid grassland microcosm. Different lower- and upper-case letters indicate significant differences (*p* < 0.05) among different nitrogen addition and plant diversity treatment, respectively (Tukey’s HSD) in semi-arid grassland mesocosms. ns indicates that the difference is not significant. S1: 1 species, S3: 3 species, S6: 6 species. N0: without nitrogen addition, N1: 5 g N m^−2^ yr.^−1^, N2: 10 g N m^−2^ yr.^−1^.

**Figure 4:**
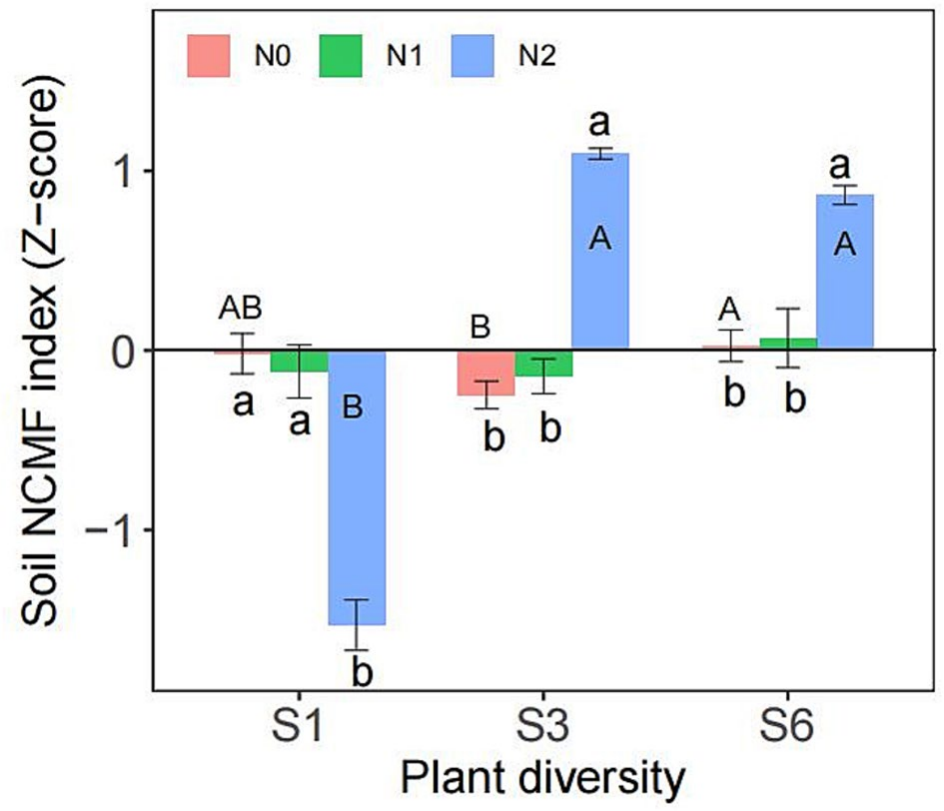
Responses of soil NCMF index to nitrogen addition and plant diversity. Different lower- and upper-case letters indicate significant differences (*p* < 0.05) among different nitrogen addition and plant diversity treatment, respectively (Tukey’s HSD) in semi-arid grassland mesocosms. S1: 1 species, S3: 3 species, S6: 6 species. N0: without nitrogen addition, N1: 5 g N m^−2^ yr.^−1^, N2: 10 g N m^−2^ yr.^−1^.

Two-way ANOVA showed that both plant diversity and nitrogen addition significantly influenced the abundances of *nifH*, *nirK*, *nirS* (Supplementary Table S1). Additionally, the interaction between plant diversity and nitrogen addition significantly affected on the abundances of *nifH*, AOB *amoA*, *nirK*, *nirS*, R_m_, R_n_, and soil NCMF (Supplementary Tables S1, S2). The abundance of *nifH* was significantly lower in the N2 treatment than in the N0 and N1 treatments (Figure 3A). Under the S1 condition, the N2 treatment significantly reduced the abundance of AOB *amoA*, *nirK*, *nirS*, and soil NCMF (Figures 3B–D, 4). However, under the S3 and S6 conditions, N2 significantly increased their abundance and soil NCMF (Figure 4). Under the N2 condition, S3 and S6 treatment significantly increased the abundances of *nifH*, AOB *amoA*, *nirK*, *nirS*, R_m_, and R_n_ than the S1 treatment (Figures 3A–F). Under N1 and N2 condition, S3 and S6 treatment significantly increased the soil NCMF than the S1 treatment (Figure 4).

### 3.5 Indirect effects of plant diversity and nitrogen addition on soil NCMF

**Figure 5:**
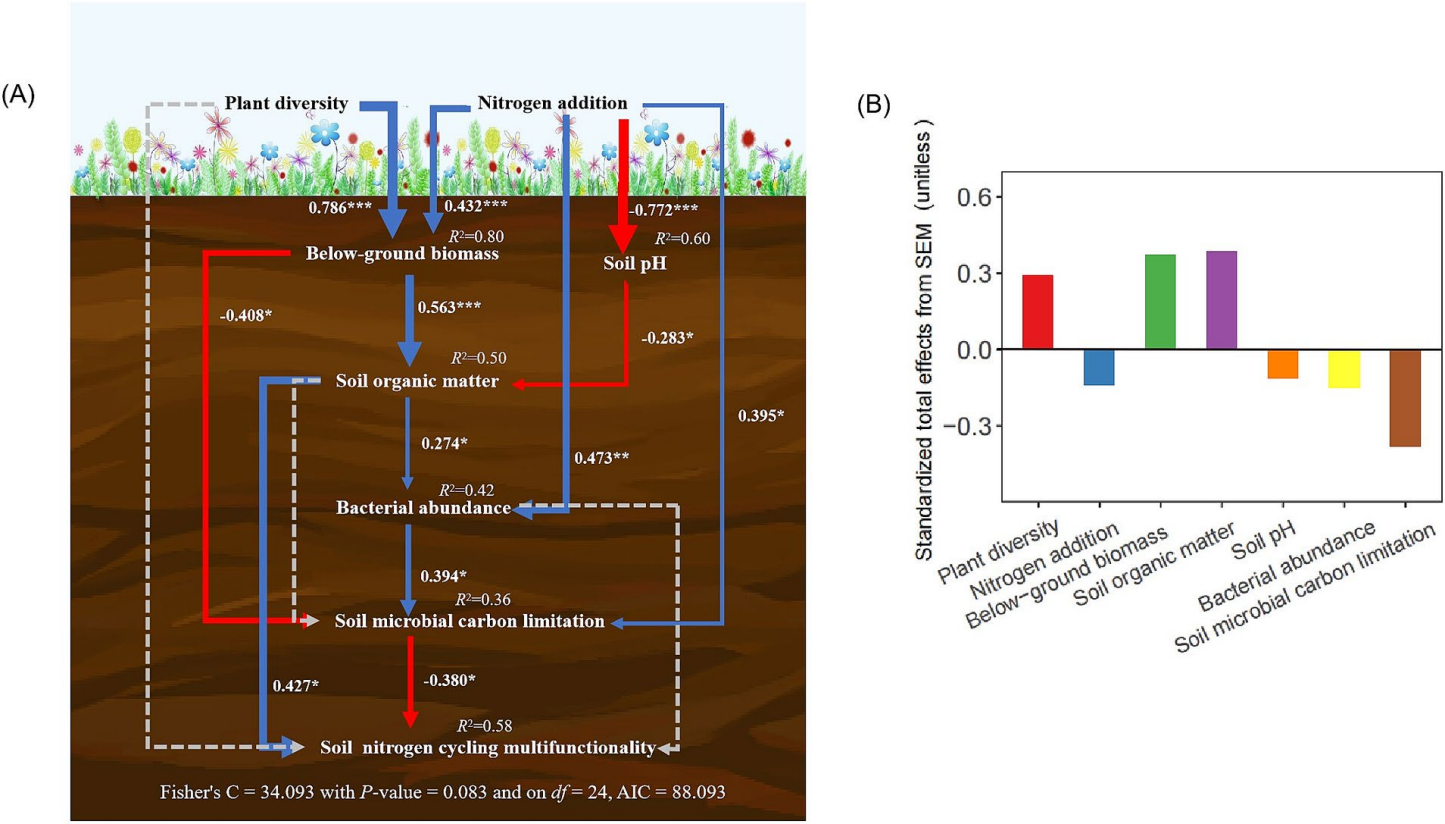
The piecewise structural equation model was used to test the direct and indirect causality between plant diversity, nitrogen addition, and soil NCMF **(A)**. The corresponding values of the solid line arrows are the width of normalized path system arrows reflect the size of normalized path coefficient. The blue and red arrows show significant positive and negative correlations, respectively (^*^p < 0.05, ^**^p < 0.01 and ^***^p < 0.001). Dashed lines show non-significant relationships. The values above each variable represents the explanatory degree (*R*^2^) of each variable in the model. Standardized total effects (direct plus indirect effects) derived from the piecewise structural equation model **(B)**.

Piecewise structural equation modeling (SEM) demonstrated that soil NCMF was indirectly influenced by plant diversity and nitrogen addition through pathways involving BGB, soil pH, SOM, bacterial abundance, and microbial carbon limitation (Figure 5A). Plant diversity alleviated the negative effects of nitrogen addition on soil NCMF by increasing BGB and SOM, which reduced microbial carbon limitation and mitigated the pH reduction caused by nitrogen addition (Figure 5A). SOM displayed largest positive effects on soil NCMF (Figure 5B). However, soil microbial carbon limitation exhibited a highest negative effect on soil NCMF (Figure 5B).

## 4 Discussion

Our findings indicate that plant diversity and nitrogen addition interactively influence soil NCMF (Table 2), suggesting that the impact of nitrogen addition depends on the diversity of the plant community (Figure 4). Specifically, nitrogen addition significantly increased the soil NCMF under the S3 and S6 conditions but not under the S1 condition (Figure 4). This implies that greater plant diversity can mitigate the adverse effects of nitrogen addition on soil NCMF. In more diverse plant communities, nitrogen addition disrupts soil nitrogen cycling less severely than it does in less diverse communities.

**Table 2:** Two-way ANOVA testing the effects of plant diversity (S), nitrogen addition (N), and their interaction (S × N) on soil NCMF and soil microbial carbon limitation in semi-arid grassland microcosm.

	S			N			S × N		
	*df*	*F*	*P*	*df*	*F*	*P*	*df*	*F*	*P*
Soil NCMF	2	2.01	0.15	2	66.37	**<0.001**	4	67.35	**<0.001**
Soil microbial carbon limitation	2	0.43	0.655	2	18.78	**<0.001**	4	6.50	**<0.001**

Significant *p* values (*p* < 0.05) are shown in bold.

### 4.1 Effects of plant diversity and nitrogen addition on soil NCMF

This study showed that nitrogen addition decreased the soil NCMF under low-diversity conditions (Figure 4); however, under high-diversity conditions, nitrogen addition significantly increased the soil NCMF, and the negative effects resulting from nitrogen addition in the low-diversity treatments were counteracted (Figure 4). The SEM clearly explained these effects (Figure 5A). Nitrogen enrichment can substantially alter soil properties, such as soil acidification (Table 1) and losses of base cations (Matson et al., 1999; Peñuelas et al., 2013). Furthermore, soil acidification-induced reductions in soil nitrogen cycling microbial activity and organic matter decomposition rates might overwhelm the positive effects of increased plant productivity under nitrogen addition (Song et al., 2021). Furthermore, the Songnen grassland in the semi-arid area was greatly limited by nitrogen (Shi et al., 2021); thus, improved nitrogen availability and supplies might have shifted soil nitrogen cycling microbes from nitrogen limitation to carbon limitation (Zhang et al., 2021). Finally, these decreases in nitrogen cycling function gene abundances have been known to be drivers of the decline in the soil nitrogen transformation rate (Ning et al., 2015; Tang et al., 2018), which likely caused the observed decrease in soil NCMF. This result is consistent with a previous study reporting that soil acidification may impede the N cycling process with nitrogen deposition (Tian and Niu, 2015).

As we hypothesized, the responses of the soil NCMF to nitrogen deposition were modified by plant diversity (Figures 4, 5). The SEM results showed that high plant diversity could offset the high nitrogen-induced decrease in soil NCMF through direct and indirect effects compared with low plant diversity (Figure 5), suggesting that plant diversity could significantly maintain and regulate nitrogen cycling in a grassland ecosystem. The direct pathway might be regulated by the changes in nitrogen concentration in roots, thus directly affecting soil nitrogen transformations because of the positive correlation between the nitrogen concentrations in roots and the rate of soil nitrogen turnover (Mueller et al., 2013).

The increase in plant below-ground biomass may be another possible mechanism by which high plant diversity offsets the negative effect of nitrogen addition on soil NCMF. It is well known that the input of plant below-ground biomass is an essential source of the grassland soil carbon pool (Reich et al., 2001; Li et al., 2017; Li et al., 2021). The present study found that high plant species diversity increased below-ground biomass, which is consistent with the previous results that there was a positive correlation between plant diversity and productivity (Reich et al., 2001). The increased soil carbon resources provided rich substrates and energy for microorganism growth and basal metabolism so that the abundance of nitrogen cycling genes and the extracellular enzyme activities associated with nitrogen cycling significantly increased, thus offsetting the negative effect of high nitrogen addition on soil NCMF under low diversity. The enhancement of soil enzyme activity in this study could be partly attributed to the increase in plant below-ground biomass (Table 1). Plant roots are not only the primary organs for nutrient uptake but also significant sources of soil extracellular enzymes. Under higher plant diversity communities, the root systems become more complex and extensive, leading to a higher input of root exudates into the soil (Reich et al., 2001; Li et al., 2017). These root exudates, which include organic acids, sugars, and amino acids, can directly promote the activity of soil extracellular enzymes (Yuan et al., 2020; Liu et al., 2021). Therefore, the impact of plant diversity on soil NCMF is not solely by the secretion of extracellular enzymes by microorganisms but also by the mediation of plant roots and their exudates. The relative abundance of corresponding nitrogen cycling functional genes has been demonstrated to regulate nitrogen transformation processes (Li et al., 2020a). For example, we found that nitrogen addition strongly increased the abundances of the AOB *amoA*, *nirK*, and *nirS* genes under high plant diversity conditions (Figure 3). Ammonia oxidation is the first rate-limiting step of nitrification that involves converting from ammonia to nitrite (Tang et al., 2016). In this study, the increased AOB *amoA* genes might accelerate the net nitrogen nitrification rate (Kowalchuk and Stephen, 2001; Osburn et al., 2021) and then enhance the soil NCMF (Figure 4C). These results supported our hypothesis, suggesting that higher plant diversity might benefit grassland ecosystem multifunctionality (Wang et al., 2019; Moi et al., 2021).

### 4.2 Interactive effects of plant diversity and nitrogen addition on soil NCMF

The observed interaction can be attributed to several underlying mechanisms. First, nitrogen enrichment can significantly alter soil properties (Peñuelas et al., 2013; Elrys et al., 2021), such as by inducing soil acidification (Table 1). A lower soil pH can directly inhibit the growth and activity of soil bacteria, which are often less tolerant of acidic conditions than fungi are (Zhang et al., 2008). Acidic conditions may favor fungal dominance over bacterial dominance (Hu et al., 2024). While fungi are generally more acid tolerant, they may decompose organic matter more slowly than bacteria do, potentially reducing overall nutrient cycling rates (Wang et al., 2011; Wang et al., 2023). Key nitrogen-cycling microbes, such as nitrifiers, are particularly sensitive to soil pH changes, and their reduced activity under acidic conditions can slow nitrogen transformation processes, such as nitrification and denitrification (Wu et al., 2024). Soil acidification can decrease the solubility of soil organic carbon, decreasing its availability to microbes, which can exacerbate microbial carbon limitations (Figure 1E), particularly in ecosystems where carbon availability is already low (Hallin et al., 2009; Cui et al., 2020; Cui et al., 2021; Song et al., 2021). Carbon-limited microbes may exhibit reduced enzymatic activity, slowing the decomposition of soil organic matter and the release of nutrients (Shu et al., 2011; Kivlin and Treseder, 2013). In some cases, high nitrogen availability can stimulate microbial activity, leading to faster decomposition of organic matter (Fornara and Tilman, 2008). However, carbon inputs (e.g., BGB) do not keep pace, which can deplete soil carbon stocks over time. In the nitrogen-limited Songnen grasslands of semi-arid regions (Shi et al., 2021), improved nitrogen availability may shift soil microbial communities from nitrogen limitation to carbon limitation (Zhang et al., 2008). Under soil carbon limitation, microbes may allocate more resources to acquire carbon (e.g., producing extracellular enzymes such as βG) (Figure 1C), potentially altering nutrient cycling dynamics (Hu et al., 2024).

Second, plant diversity may counteract microbial carbon limitation by increasing BGB (Figure 5). Diverse root structures and carbon-rich root exudates, along with increased organic matter from root turnover, provide additional carbon sources for soil microbes, alleviating carbon limitations (Yuan et al., 2020; Liu et al., 2021). Higher plant diversity leads to increased root turnover, contributing to SOM accumulation and providing a continuous supply of carbon for microbial communities (Figure 5) (Freitag et al., 2023). Furthermore, diverse plant communities may utilize nitrogen more efficiently, reducing soil acidification. Increased SOM not only supplies carbon but also enhances soil structure, water retention (Table 1), and microbial habitats (Chen et al., 2020; Cheng et al., 2022). This enhanced microbial activity can improve organic matter decomposition, increasing the amount of carbon released into the soil (Chen and Chen, 2021). With sufficient carbon, microbes can process nitrogen more efficiently (Tang et al., 2016), potentially increasing soil net nitrogen mineralization and nitrification rates. In diverse plant communities, species may utilize nitrogen in different ways or at different times, leading to more efficient nitrogen use overall (Maestre et al., 2011; Zhang et al., 2021; Xu et al., 2022). This reduces excess soil nitrogen, mitigating the negative effects of nitrogen addition. For example, deep-rooted and shallow-rooted plants may access nitrogen from different soil layers, preventing nitrogen leaching (Oelmann et al., 2011). The insurance hypothesis further suggests that diverse communities are more resilient to stressors such as excess nitrogen, as compensatory mechanisms among species stabilize ecosystem functions, including nitrogen cycling (Xu et al., 2022). Diverse plant communities may also increase soil microbial abundance (Figures 2A,B), fostering robust nitrogen cycling processes that can accommodate added nitrogen without becoming unbalanced (Lucas-Borja and Delgado-Baquerizo, 2019; Wang et al., 2023). This creates a positive feedback loop, as healthy nitrogen cycling supports plant growth. The buffering effect of plant diversity may depend on functional diversity rather than species richness alone (Liu et al., 2021). For example, legumes, which fix atmospheric nitrogen, may interact differently with added nitrogen than nonlegumes do. Future research should explore these interactions further (Suter et al., 2015; Singh et al., 2018).

### 4.3 Implications for ecosystem management and climate change mitigation

These findings have significant implications for ecosystem management and climate change mitigation. In the context of global nitrogen deposition and the increasing use of nitrogen fertilizers, understanding the role of plant diversity in regulating soil NCMF is essential for predicting soil nitrogen dynamics and formulating sustainable land management strategies. Our results demonstrate that maintaining or enhancing plant diversity in terrestrial ecosystems can serve as a buffer against the adverse effects of nitrogen addition on soil NCMF. This study not only underscores the interactive effects of plant diversity and nitrogen addition on soil NCMF but also elucidates the underlying mechanisms driving these interactions. These insights highlight the critical need to incorporate plant diversity into strategies aimed at managing soil nitrogen dynamics amidst changing nitrogen regimes. Future research should focus on investigating the long-term impacts of plant diversity on soil NCMF, as well as the potential feedback mechanisms between soil nitrogen cycling and ecosystem productivity across diverse environmental scenarios. Such efforts will further refine our understanding of ecosystem resilience and inform more effective management practices in the face of global environmental change.

## 5 Conclusion

Our study provides robust evidence that plant diversity plays a pivotal role in mitigating the adverse effects of nitrogen addition to soil NCMF. Specifically, we demonstrate that the interactive effects of plant diversity and nitrogen addition on soil NCMF are mediated through a combination of indirect pathways, including changes in BGB, SOM, soil pH, bacterial abundance, and microbial carbon limitation. These mechanisms collectively highlight the multifaceted ways in which plant diversity buffers the negative impacts of nitrogen enrichment on soil nitrogen cycling processes. These findings provide novel insights into the mechanisms by which plant diversity and nitrogen addition influence soil NCMF in semi-arid grasslands, offering valuable implications for the understanding and sustainable management of grassland ecosystems under global climate change.

## Data Availability

The raw data supporting the conclusions of this article will be made available by the authors, without undue reservation.
