# Supplementary material for: High plant diversity alleviates the negative effects of nitrogen deposition on soil nitrogen cycling multifunctionality
Source: Front Microbiol. 2025 May 14;16:1596515. doi: 10.3389/fmicb.2025.1596515 (PMC12116467; doi:10.3389/fmicb.2025.1596515)
Supplement: Supplementary file 1 [file Data_Sheet_1.docx]

Supplementary Material

**Table S1** Two-way ANOVA testing the effects of plant diversity (S), nitrogen addition (N), and their interaction (S × N) on plant and soil parameters in semi-arid grassland mesocosms. Significant *P* values (*P* < 0.05) are shown in bold.

|  | S | | |  |  | N |  |  | S × N | | |
| --- | --- | --- | --- | --- | --- | --- | --- | --- | --- | --- | --- |
|  | *df* | *F* | *P* |  | *df* | *F* | *P* |  | *df* | *F* | *P* |
| pH | 2 | 4.671 | **0.02** |  | 2 | 2.277 | 0.12 |  | 4 | 18.413 | **<0.001** |
| SWC | 2 | 6.278 | **0.005** |  | 2 | 3.49 | **0.043** |  | 4 | 30.529 | **<0.001** |
| TC | 2 | 7.9 | **0.002** |  | 2 | 4.78 | **0.02** |  | 4 | 24.427 | **<0.001** |
| SOM | 2 | 3.562 | **0.040** |  | 2 | 1.597 | 0.218 |  | 4 | 2.344 | 0.075 |
| AGB | 2 | 10.772 | **<0.001** |  | 2 | 6.844 | **0.003** |  | 4 | 0.962 | 0.441 |
| BGB | 2 | 24.622 | **<0.001** |  | 2 | 6.173 | **0.005** |  | 4 | 2.521 | 0.06 |
| NO_3_^–^–N | 2 | 0.063 | 0.939 |  | 2 | 41.784 | **<0.001** |  | 4 | 12.2 | **<0.001** |
| NH_4_^+^–N | 2 | 31.77 | **<0.001** |  | 2 | 29.157 | **<0.001** |  | 4 | 59.412 | **<0.001** |
| TN | 2 | 22.495 | **<0.001** |  | 2 | 221.166 | **<0.001** |  | 4 | 148.458 | **<0.001** |
| *nifH* | 2 | 27.001 | **<0.001** |  | 2 | 208.13 | **<0.001** |  | 4 | 36.746 | **<0.001** |
| AOB *amoA* | 2 | 2.011 | 0.15 |  | 2 | 77.926 | **<0.001** |  | 4 | 104.912 | **<0.001** |
| *nirK* | 2 | 4.36 | **0.021** |  | 2 | 17.724 | **<0.001** |  | 4 | 29.164 | **<0.001** |
| *nirS* | 2 | 3.42 | **0.045** |  | 2 | 21.964 | **<0.001** |  | 4 | 51.375 | **<0.001** |
| R_m_ | 2 | 6.895 | **<0.01** |  | 2 | 0.908 | 0.413 |  | 4 | 4.58 | **<0.01** |
| R_n_ | 2 | 6.693 | **<0.01** |  | 2 | 0.81 | 0.454 |  | 4 | 4.003 | **<0.01** |
| NAG | 2 | 2.233 | 0.124 |  | 2 | 19.226 | **<0.001** |  | 4 | 48.28 | **<0.001** |
| LAP | 2 | 20.322 | **<0.001** |  | 2 | 7.201 | **0.003** |  | 4 | 11.524 | **<0.001** |
| βG | 2 | 2.913 | 0.068 |  | 2 | 4.513 | **0.018** |  | 4 | 11.671 | **<0.001** |
| ALP | 2 | 3.671 | **0.036** |  | 2 | 4.93 | **0.013** |  | 4 | 5.078 | **0.002** |
| Bacterial abundance | 2 | 8.591 | **0.001** |  | 2 | 16.377 | **<0.001** |  | 4 | 8.175 | **<0.001** |
| Fungal abundance | 2 | 0.256 | 0.775 |  | 2 | 0.069 | 0.932 |  | 4 | 0.539 | 0.707 |
| fungi to bacteria ratio | 2 | 0.703 | 0.502 |  | 2 | 0.635 | 0.536 |  | 4 | 1.160 | 0.346 |
| Microbial biomass carbon | 2 | 7.243 | **0.002** |  | 2 | 2.906 | 0.069 |  | 4 | 4.687 | **0.004** |


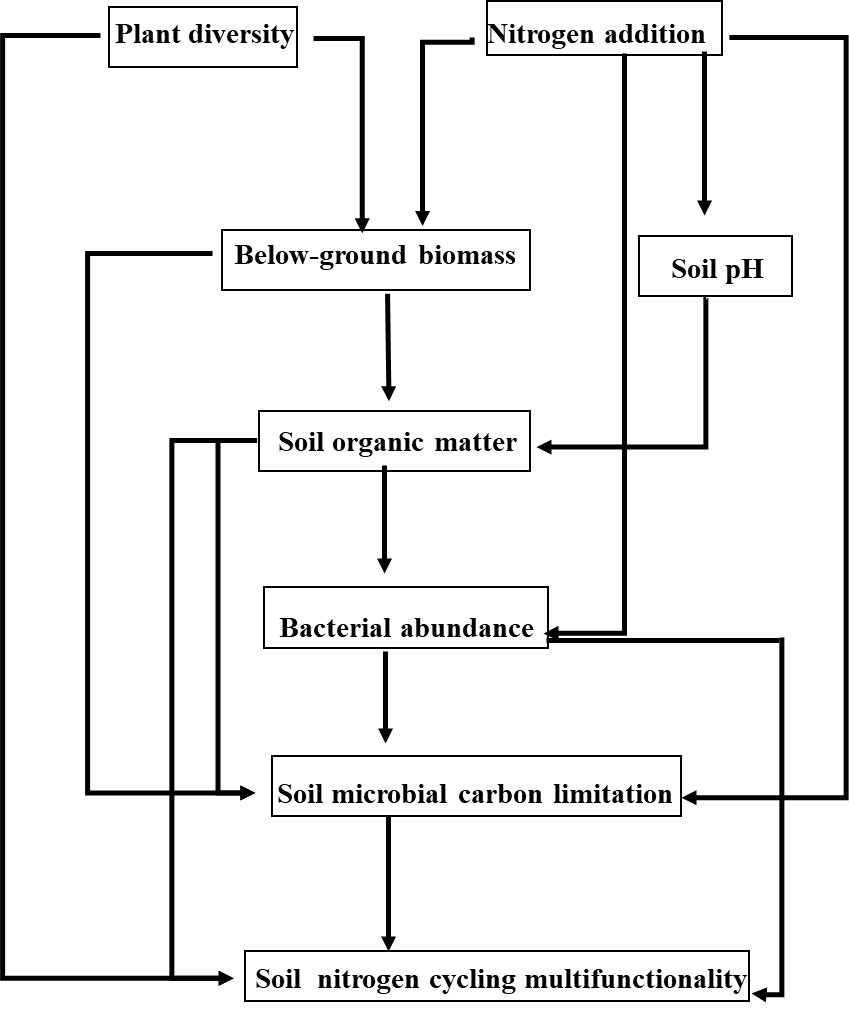


**Figure S1.** A *prior* conceptual model of how nitrogen addition and plant diversity altered soil NCMF. The model contains six hierarchical pathways that cause changes in the soil NCMF: 1) the direct pathways of nitrogen addition and plant diversity on soil pH and below-ground biomass; 2) the indirect pathways of nitrogen addition and plant diversity on soil organic matter; 3) the indirect pathways of nitrogen addition and plant diversity on soil bacterial abundance; and 4) the indirect pathways of nitrogen addition and plant diversity on soil NCMF via changes in soil microbial carbon limitation.
